# Supplementary material for: HIVprotI: an integrated web based platform for prediction and design of HIV proteins inhibitors
Source: J Cheminform. 2018 Mar 9;10:12. doi: 10.1186/s13321-018-0266-y (PMC5845081; doi:10.1186/s13321-018-0266-y)
Supplement: Supplementary file 2 — Additional file 2. Source code of HIVProtI web server. [file 13321_2018_266_MOESM2_ESM.zip › HIVprotI_Source-code/hivprotI/weka-3-6-6/doc/weka/gui/visualize/PrintableHandler.html]

PrintableHandler


---


|  |  |  |
| --- | --- | --- |
| **Overview** \| **Package** \| **Class** \| **Tree** \| **Deprecated** \| **Index** \| **Help** \| **Weka's home** | |  |
| **PREV CLASS**   **NEXT CLASS** | **FRAMES**    **NO FRAMES**     **All Classes** |  |
| SUMMARY: NESTED \| FIELD \| CONSTR \| METHOD | DETAIL: FIELD \| CONSTR \| METHOD |  |


---


## weka.gui.visualize Interface PrintableHandler

**All Known Implementing Classes:**: AttributeVisualizationPanel, HierarchyVisualizer, PrintableComponent, PrintablePanel, ThresholdVisualizePanel, TreeVisualizer, VisualizePanel

---

``` public interface PrintableHandler ```

This interface is for all JComponent classes that provide the ability to
print itself to a file.

**Version:**
:   $Revision: 1.2 $

**Author:**
:   FracPete (fracpete at waikato dot ac dot nz)

**See Also:**: `PrintableComponent`, `PrintablePanel`

---

| **Method Summary** | |
| --- | --- |
| `java.lang.String` | `getSaveDialogTitle()`             returns the title for the save dialog |
| `JComponentWriter` | `getWriter(java.lang.String name)`             returns the JComponentWriter associated with the given name, is `null` if not found |
| `java.util.Hashtable` | `getWriters()`             returns a Hashtable with the current available JComponentWriters in the save dialog. |
| `double` | `getXScale()`             returns the scale factor for the x-axis |
| `double` | `getYScale()`             returns the scale factor for the y-axis |
| `void` | `saveComponent()`             displays a save dialog for saving the component to a file. |
| `void` | `setSaveDialogTitle(java.lang.String title)`             sets the title for the save dialog |
| `void` | `setScale(double x, double y)`             sets the scale factor |

**Method Detail**

### getWriters

```
java.util.Hashtable getWriters()
```

:   returns a Hashtable with the current available JComponentWriters in the
    save dialog. the key of the Hashtable is the description of the writer.

    :   **Returns:**: all currently available JComponentWriters **See Also:**: `JComponentWriter.getDescription()`

---


### getWriter

```
JComponentWriter getWriter(java.lang.String name)
```

:   returns the JComponentWriter associated with the given name, is
    `null` if not found

    :   **Returns:**: the writer associated with the given name **See Also:**: `JComponentWriter.getDescription()`

---


### setSaveDialogTitle

```
void setSaveDialogTitle(java.lang.String title)
```

:   sets the title for the save dialog

---


### getSaveDialogTitle

```
java.lang.String getSaveDialogTitle()
```

:   returns the title for the save dialog

---


### setScale

```
void setScale(double x,
              double y)
```

:   sets the scale factor

    :   **Parameters:**: `x` - the scale factor for the x-axis: `y` - the scale factor for the y-axis

---


### getXScale

```
double getXScale()
```

:   returns the scale factor for the x-axis

---


### getYScale

```
double getYScale()
```

:   returns the scale factor for the y-axis

---


### saveComponent

```
void saveComponent()
```

:   displays a save dialog for saving the component to a file.


---


|  |  |  |
| --- | --- | --- |
| **Overview** \| **Package** \| **Class** \| **Tree** \| **Deprecated** \| **Index** \| **Help** \| **Weka's home** | |  |
| **PREV CLASS**   **NEXT CLASS** | **FRAMES**    **NO FRAMES**     **All Classes** |  |
| SUMMARY: NESTED \| FIELD \| CONSTR \| METHOD | DETAIL: FIELD \| CONSTR \| METHOD |  |


---
